# Investigation of the relationship between inherited thrombophilia and novel coronavirus pneumonia

**DOI:** 10.2217/fvl-2020-0395

**Published:** 2021-04-21

**Authors:** Aslihan Kiraz, Seda Guzeldag, Esma Eren, Musa Goksu, Arslan Bayram

**Affiliations:** 1 ^1^Department of Medical Genetics, Kayseri City Education & Research Hospital, Kayseri 38080, Turkey; 2 ^2^Department of Intensive Care Unit, Kayseri City Education & Research Hospital, Kayseri 38080, Turkey; 3 ^3^Department of Infectious Diseases & Clinical Microbiology, Kayseri City Education & Research Hospital, Kayseri 38080, Turkey; 4 ^4^Department of Medical Genetics, Etlik Zübeyde Hanim Women’s Diseases Education & Research Hospital, Ankara 06010, Turkey

**Keywords:** COVID-19, *F2*, *F5*, NCP, *PAI-1*, thrombophilia

## Abstract

**Aim:** This study aimed to investigate the relationship between severe novel coronavirus pneumonia (NCP) and hypercoagulable conditions that predispose patients to thrombosis such as the prothrombin gene (*F2*) rs1799963 (G20210A), factor V Leiden (*F5*) rs6025 (G1691A) and *PAI-1* (rs1799768). **Patients:** NCP-diagnosed 62 previously healthy patients were enrolled for the investigation of the thrombophilia-related polymorphisms. **Materials & methods:** The frequency of genotypes were compared with healthy control group frequencies from other studies. **Results:** There were no statistically significant differences between the severe patient group and the healthy population regarding the investigated single nucleotide polymorphisms (SNPs). **Conclusion:** This study is the first to rule out the relationship of rs1799963, rs6025 and rs1799768 with severe NCP.

Novel coronavirus pneumonia (NCP; COVID-19) is a disease caused by the enveloped viral pathogen SARS-coronavirus 2. NCP, which is a major health problem worldwide, still has no definitive treatment. Acute respiratory distress syndrome and sepsis are the main complications of the disease. Additionally, hypercoagulability mechanism similar to disseminated intravascular coagulation is one of the main underlying causes of death among patients [1,2]. A high number of thrombotic complications exist, and the incidence of thrombotic disease in individuals affected by NCP is reported to be 31% [3]. The brain and lung are affected by the hypercoagulable state, and anticoagulant therapy should be started in these NCP patients [4].

Thrombophilia is a hypercoagulable condition that predisposes patients to thrombosis. Thrombophilia is a multifactorial condition that can result from genetic factors, acquired factors or a combination of both. The prothrombin gene (*F2*) rs1799963 variant known as G20210A, factor V Leiden (*F5*) rs6025 variant known as G1691A and *PAI-1* rs1799768 variant are important polymorphic biomarkers of thrombophilia. Patients with multiple gene defects have a high risk of thrombosis [5]. Despite the hypercoagulation complications observed in NCP patients, there have been no previous investigations in terms of genetic thrombophilia predisposition.

Investigated SNPs within this study are the most commonly tested ones in different situations like cerebrovascular embolism, recurrent pulmonary thromboembolism, deep venous thrombosis, recurrent pregnancy loss, before administration of some drugs etc. Even these SNPs are combined in the ‘Thrombophilia panel’ and are commercially available and accessible in most hospitals. So, with this study, we tried to answer a question: is there a need for a ‘Thrombophilia panel genetic investigation’ in NCP diagnosed intensive care unit patients? So, by investigating selected thrombophilia factors that are thought to have a possible impact on the clinical course of the disease, we aimed to demonstrate the relationship between severe NCP and familial thrombophilia factors.

## Material & method

Adult (>18 years), previously healthy male patients treated for NCP, without any chronic disease who had severe complications, were enrolled in this study to investigate well-known thrombophilia-related polymorphisms in factor II (prothrombin), factor V (*F5*) and the *PAI-1* gene. The diagnosis of NCP was made according to the WHO interim guidance [6] and confirmed by RNA detection. The main exclusion criteria were a negative PCR result. Mild or moderate cases also were excluded who had less severe clinical symptoms such as low-grade fever and cough with no evidence of severe pneumonia. Included severe NCP patients’ criteria was a respiratory rate >30 breaths/minute, respiratory distress and oxygen saturation (SpO_2_) <93%.

The study was approved by the local institutional ethics committee in accordance with the ethical standards of the institutional and/or national research committee and with the 1964 Helsinki declaration and its later amendments or comparable ethical standards.

Blood samples taken from individuals were collected into tubes with EDTA and kept at 4°C until DNA isolation. DNA isolation was performed on the MagPurix I12 (Zinext Life Science coop., New Taipei, Taiwan) device with the MAgPurix (Zinext Life Science coop.) whole blood extraction kit. After DNA isolation (20–30 ng/μl concentration), measurements were made in a Nanodrop (Thermo Scientific, MA, USA) for quality. The ratio of absorbance at 260 and 280 nm is used to assess the purity of DNA. A ratio of ∼1.8 is accepted as ‘pure’ DNA.

PCR procedures were performed for amplification after DNA isolation from the blood of individuals. Polymorphisms were investigated using *PAI-1* real-time Hb-FRT, factor II real-time HD-FRT and factor V Leiden real-time HD-FRT kits (DIAGEN Biotechnological Systems Inc., Ankara, Turkey) by real-time PCR using the Qiagen Rotor Gene (Qiagen, Hilden, Germany) device. All testing was performed in the Detagen Genetic Diagnosis, Research And Application Center Inc. Kayseri, Turkey. The analysis was performed by using the Rotor Gene Q series software (Qiagen).

The frequency of genotypes was compared with healthy control group frequencies from other studies performed in a Turkish population [7–9], which is the best suitable data as it is the same population with the study group from the current study. For comparison of groups, the chi-square test was performed by using IBM SPSS statistics 22 software with a p-value of less than 0.05 considered statistically significant.

## Result

**Figure 1.**
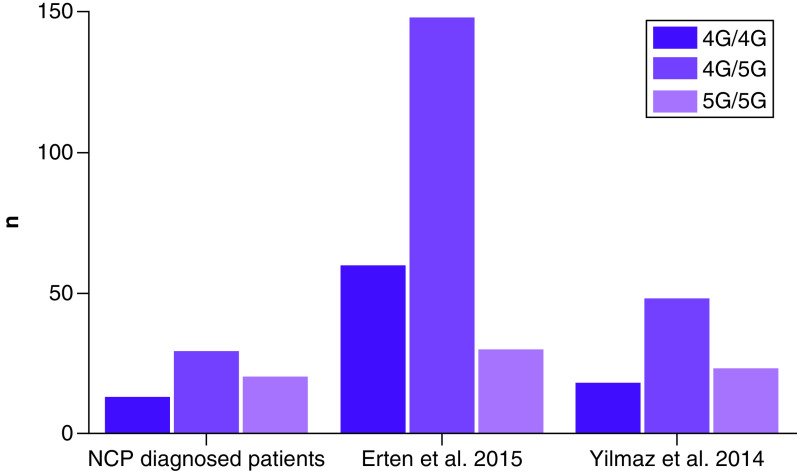
rs1799768 (*PAI-1*) ratio within groups. No significant difference between groups in the prevalence of 4G/4G genotype which is correlated with increased thrombosis risk was observed.

62 patients who met the criteria during the study were included in the study. The mean (± standard deviation) of age was 38.83 ± 11.04 (minimum 18, maximum 60) years. There was no significant difference between groups regarding the prevalence of rs1799963 (*F2*) and rs6025 (*F5* Leiden) (Table 1). While there was a statistically significant difference between groups in the prevalence of rs1799768 (*PAI-1* it was considered a coincidence and nonimportant finding because of the small sample size; upon further review, it was observed that the main cause of the difference between groups was the low prevalence of 5G/5G (12.6%) in one of the control groups [7], which did not support our hypothesis (Figure 1). There was no significant difference between groups in the prevalence of the 4G/4G genotype.

**Table 1.** Genotype frequency comparison between groups via chi-square test.

	*F2* (rs1799963)			p-value	*F5* (rs6025)			p-value	*PAI-1* (rs1799768)			p-value	Ref.
	WT	Het	Hom		WT	Het	Hom		4G/4G	4G/5G	5G/5G		
NCP diagnosed patients (n = 62)	59 (95%)	3 (5%)	0	0.66^†^	56 (90.3%)	5 (8%)	1 (1.7%)	0.057^†^	13^a,b^ (21%)	29^b^ (46.8%)	20^a^ (32.3%)	0.003	
Erten *et al.* 2015 (n = 238)	228 (95.8%)	10 (4.2%)	0		217 (91.2%)	21 (8.8%)	0		60^a^ (25.2%)	148^a^ (62.2%)	30^b^ (12.6%)		[7]
Yılmaz *et al.* 2014 (n = 89)	86 (96.6%)	3 (3.4%)	0		–	–	–		18^a^ (20.2%)	48^a^ (53.9%)	23^a^ (25.8%)		[8]
Şahin *et al.* 2012 (n = 109)	106 (97.2%)	3 (2.8%)	0		90 (82.6%)	16 (14.7%)	3 (2.7%)		–	–	–		[9]

Each superscript letter (a, b) denotes a subset of groups whose column proportions do not differ significantly from each other at the 0.05 level. For *PAI-1* (rs1799768) p < 0.05, but it was not due to the increased percentage of 4G/4G variant ratio, as shown in the table there is not significant proportion difference between 4G/4G variant (13^a, b^ [21%]) and 5G/5G (20^a^ [32.3%]) and 4G/5G (29^b^ [46.8%]) variant proportions.

† Heterozygous and homozygous values combined due to some of the values not being greater than 1.

## Discussion

The risk of thrombosis and arterial and venous thromboembolic complications seen in 30% of hospitalized subjects due to NCP has been reported in many studies, which can be explained by the prolonged inflammatory response, decreased physical activity during infection and reduced oxygen levels in the circulation. Some reports raise the alarm regarding this complication, such as increased thromboembolism incidence despite prophylaxis [10]; a sevenfold increase in large vessel stroke in some patients who experienced either no or mild COVID-19 symptoms [11]; and, cerebral infarct occurrence in NCP diagnosed patients with thrombocytopenia, coagulopathy and increased anticardiolipin antibodies [12], which is very worrying and needs further investigation of the molecular basis of this phenomenon.

In the literature, lung thromboembolism as well as a thrombus in different localizations have been reported in NCP cases [13,14]. Fox *et al.* also detected fibrin thrombi in small vessels and capillaries and argued that it would be beneficial to use agents that also treat thrombotic and microangiopathic effects caused by the virus [15].

According to the observations of some physicians in Turkey, it is suspected that genetic factors are affecting the course of NCP because of cases that are relatives and have similar severe clinical complications despite living in different provinces. Again, in these observations, the presence of young people who died at a young age and with a severe NCP was observed, although they did not have any underlying medical conditions.

Clinical observation of the intensive care unit physicians from all over the world indicates that NCP-diagnosed patients are very hypercoagulable and that there is a high rate of micro-pulmonary embolism between patients. Likely, some patients already had pulmonary embolism before hospitalization who were not responsive to prophylactic doses of heparins during their hospital stay [16]. Additionally, many other studies [17–20] indicate that abnormal coagulation results are common (especially elevated D-dimer) in NCP-related deaths. Considering all of the abovementioned factors, it is possible that having a genetic predisposition to thrombophilia would be a possible susceptibility factor for severe NCP.

There are many studies [21] showing relationships between genetic thrombophilia factors (*F5*, *PAI-1*, etc.) and thromboembolic disease, which is frequently seen in severe NCP and is often associated with mortality [22]. Gralinski *et al.* investigated the viral pathogenesis of SARS-coronavirus disease and suggested that *PAI-1* plays a protective role against infection [23].

According to Berri *et al.* [24], 6-aminocaproic acid can protect against influenza, as plasminogen contributes to inflammation caused by influenza. The application of aggressive anticoagulation by using inexpensive antithrombotic drugs is very attractive [16], but finding genetic markers before using them is essential, and such markers would be very important for the determination of people who are susceptible to severe NCP primary infection.

During disasters, government organizations make available all possible resources, but it is the responsibility of those who are on the front line to use resources sensibly. As there were and may be an idea to use similar gene panels in severe NCP patients, this preliminary study shows that there is no need in spending resources for testing at least SNP’s investigated in this study. However, this data considered to be sufficient in terms of planning further studies.

A limitation of this study is that it only covers specific SNPs of three genes that are the most common and routinely checked thrombophilia factors. Despite initial plans to investigate larger gene panels, due to financial support of the organization it was decided to focus on the most popular factors. Despite the small sample size, we hope this preliminary study will lead to large-scale upcoming studies that can be performed with a greater sample size covering more related genetic factors.

## Conclusion

While investigating the frequency of suspected common SNPs of *F2*, *F5* and *PAI-1* genes in this study, there were no statistically significant differences between the severe patient group and the healthy population in SNPs. Given these findings, there is a need for research focused on other thrombophilia-related genetic factors that contribute to severe NCP.

**Summary points**

- It is known that there is a relationship between severe novel coronavirus pneumonia (NCP) and hypercoagulable conditions that predispose patients to thrombosis.
- The prothrombin gene (*F2* rs1799963/G20210A), factor V Leiden (*F5* rs6025/G1691A) and *PAI-1* (rs1799768) are important polymorphic biomarkers of thrombophilia that are investigated in severe NCP patients within this study.
- There were no statistically significant differences between the severe patient group and the healthy population regarding the investigated SNPs.
- This study is the first to rule out the relationship of rs1799963, rs6025 and rs1799768 with severe NCP. There is still a need for research focused on other thrombophilia-related genetic factors that contribute to severe NCP, which may explain the difference between the clinical course of the disease.
